# The interplay of influenza and COVID-19 in Germany, January 2020 - December 2022: a study of competitive disease dynamics with quarantine measures and partial cross-immunity

**DOI:** 10.1186/s12889-025-24362-z

**Published:** 2025-09-09

**Authors:** Anna Daniel Fome, Isti Rodiah, Wolfgang Bock, Berit Lange, Axel Klar

**Affiliations:** 1 Department of Mathematics, Rheinland-Pfälzische Technische Universität Kaiserslautern-Landau, Gottlieb-Daimler-Str.48, Kaiserslautern, 67663 Germany; 2 Department of Economics, Mathematics and Statistics, Jordan University College, P.O.Box 1878, Morogoro, Tanzania; 3 Department of Epidemiology, Helmholtz Centre for Infection Research (HZI), Inhoffenstr. 7, Braunschweig, 38124 Germany; 4 Department of Mathematics, Linnaeus University, Universitetsplatsen 1, Växjö, 35252 Sweden; 5 German Centre for Infection Research (DZIF), Inhoffenstr. 7, Braunschweig, 38124 Germany

**Keywords:** Influenza, COVID-19, Age-stratified model, Multi-pathogen, Competition, Co-existence, Cross-immunity

## Abstract

We study the dynamics of coexisting influenza and SARS-CoV-2 by adapting a well-established age-specific COVID-19 model to a multi-pathogen framework. Sensitivity analysis and adjustment of the model to real-world data are used to investigate the influence of age-related factors on disease dynamics. Our findings underscore the critical role that transmission rates play in shaping the spread of influenza and COVID-19. Furthermore, our analysis highlights the significant interaction between influenza and SARS-CoV-2 transmission rates, particularly in scenarios with partial cross-immunity. This underscores the importance of comprehensive interventions that simultaneously target both pathogens to effectively control their spread in coexisting environments. Our results demonstrate the importance of age-structured models in capturing the dynamics of influenza and COVID-19, underscoring the importance of accounting for age structure. Notable disparities emerge in estimated transmission rates between single-pathogen and multi-pathogen models, shedding light on the complex interactions between pathogens and their impacts on disease transmission across different age groups.

**Supplementary Information:**

The online version contains supplementary material available at 10.1186/s12889-025-24362-z.

## Introduction

The emergence of coronavirus disease in 2019 (COVID-19) has highlighted significant global challenges for public health systems, while seasonal influenza continued to cause substantial threats, particularly in temperate regions like Germany. The cocirculation of these respiratory viruses has raised concerns about their interactions and the burden on health resources [1]. Influenza, which peaks in winter, produces marked morbidity and mortality annually, mainly among vulnerable groups [2]. In contrast, COVID-19, driven by SARS-CoV-2, has caused widespread disruptions since its appearance, mainly due to the lack of pre-existing immunity in the population, and has been further intensified by recurrent waves and the emergence of new variants [3, 4]. Unlike the predictable pattern of influenza, the impact of COVID-19 has fluctuated, shaped by public health measures and vaccination efforts [5]. Tuberculosis (TB) is another respiratory infection that contributes to the complexity of public health management, especially in regions where the prevalence of TB is increasing [6]. All of these viruses spread primarily through respiratory droplets [7], making it crucial to understand their individual and combined impacts to develop effective public health strategies and manage the dual burden on healthcare systems.

Cocirculating pathogens can interact through shared host populations, cross-immunity [8], competition for ecological niches [9], or co-infections [10]. These interactions may result in competitive exclusion or coexistence, shaped by transmission dynamics, immune response, and public health interventions [11]. Understanding such mechanisms is key to anticipating and managing outbreaks involving pathogens such as influenza and COVID-19.

To investigate these dynamics, various multi-pathogen models have extended traditional frameworks to incorporate pathogen competition, cross-immunity, and co-infection. For example, Lazebnik et al. used an extended SIRD model to show that increasing strain diversity can increase infection and mortality rates [12]. Similarly, models by Ojo et al. and Musa et al. explored the cocirculation of COVID-19 and influenza, demonstrating that combined interventions, especially vaccination, can mitigate epidemic peaks [13, 14]. The foundational work by Andreasen and Fudolig et al. analyzed the role of partial cross-immunity in driving oscillatory behavior and strain persistence [8, 15]. Waterlow et al. and Nuno et al. further highlighted the role of quarantine and cross-protection in shaping multi-pathogen outcomes [16, 17].

Influenza and COVID-19 epidemics have been effectively managed through quarantine and non-pharmaceutical interventions (NPIs) such as lockdowns, mask use, social distancing, travel restrictions, school and workplace closures, limits on public gatherings, and enhanced hygiene measures [18]. Similar strategies remain important for the control of other respiratory diseases, including tuberculosis (TB) [19]. In addition to these measures, some studies have explored the possibility that previous infections or vaccinations may provide partial cross-protection between influenza and COVID-19, which could influence disease dynamics by affecting susceptibility [17, 20]. Although some findings suggest cross-reactive immune responses, for example, shared peptides between the SARS-CoV-2 and influenza A subtypes, or enhanced neutralizing responses in animal studies, this area remains under investigation and the evidence is not yet conclusive [21–23]. Similarly, some studies have reported a possible association between influenza vaccination and a reduced severity or risk of COVID-19, although the findings vary and more research is needed [24]. Importantly, there is currently no evidence that COVID-19 vaccines protect against influenza [25, 26]. Overall, while the potential for cross-immunity is a topic of scientific interest, its role in shaping co-circulating respiratory infections is still unclear and requires further study.

Age-based models, using data from various groups, also help create effective public health strategies by estimating disease susceptibility, severity, and health care needs between populations [27, 28]. For example, the SIR (Susceptible-Infected-Recovered) framework, when combined with age-specific contact matrices, as demonstrated by Prem et al. [29], helps simulate disease transmission between age groups and assess the potential impact of interventions such as school closures and workplace distancing. Focusing on age-related vulnerability, COVID-19 has shown significant differences in how the disease affects various age groups. Some studies, such as [27, 30] investigate age-specific transmission dynamics, noting that contact patterns significantly influence transmission rates. The latter find that children and young adults have different contact patterns compared to older adults, affecting the transmission dynamics [27]. Meanwhile, [30] indicate that children, although susceptible to infection, are less likely to be significant transmission drivers compared to adults. Further studies emphasize the differential impacts of COVID-19 between age groups. Atchison et al. [31] examine long COVID symptoms, showing that younger populations experience significant long-term effects despite older adults being at increased risk of severe initial illness. Meanwhile, studies by Levin et al. [32] and Wang et al. [33] focus on the severe outcomes faced by elderly populations, including higher hospitalization and mortality rates, prolonged recovery periods, and increased complications, emphasizing the need for targeted protective measures for older individuals.

However, a thorough analysis of national influenza surveillance data from [34] shows that elderly people and children are especially susceptible to serious consequences, such as increased rates of hospitalization and mortality during the influenza seasons. By comparing historical and modern pandemics, the literature further investigates age-specific trends and demonstrates that although young people and the elderly are the most affected, children are typically the main carriers of influenza [35]. Other recent studies, for example, [36] have brought attention to the need to develop an age-specific model to improve public health responses and understanding of influenza dynamics. This will help to ensure that control measures are effectively tailored to the varying impacts across different age groups.

In our previous work [37], we presented a deterministic SEIQR (Susceptible-Exposed-Infected-Quarantined-Recovered) epidemic model in a host population with two competing pathogens, pathogen-*i* (influenza) and pathogen-*c* (COVID-19). The model includes the concepts of co-infection, cross-immunity, and quarantine and aims to comprehensively explore the dynamics of co-existence between these two pathogens under control measure. The analysis in [37] included both analytic and numerical evaluation using heuristic data, which provided a preliminary understanding of the potential performance and stability of the model in different scenarios. In this paper, we refine and validate the model with empirical data from real observations.

Regarding co-infections, a review by [38], which included studies from Asia, Europe, and the Americas, found 749 cases of respiratory viral infections among patients with COVID-19. The global prevalence of these co-infections was 5.01%, with influenza viruses (1.54%) and enteroviruses (1.32%) being the most common according to 56 studies. They concluded that, although COVID-19 is still widespread, co-infections with a respiratory virus are relatively low. For example, a clinical case in Germany involved a 4-month-old child coinfected with SARS-CoV-2 and influenza A, highlighting the importance of accurate diagnosis and treatment [39]. Similar patterns have been observed in other countries, with two cases in the United States [40], four in Spain [41], and more than 10 cases in China [42]. In addition, there were remarkably few reports of other co-infections such as influenza-RSV (respiratory syncytial virus) and Rhinovirus-SARS-CoV-2 [43] between 2020 and 2022. This indicates that although co-infections do occur, they are relatively uncommon. Therefore, the current study focuses on broader trends in viral circulation, and co-infections are not included in the model used for this analysis.

The objective of this study is to comprehensively analyze the interactions between influenza and COVID-19 in Germany from January 2020 to December 2022. By incorporating infection data and employing both age-specific and all-age SEIQR models, the study aims to provide valuable insights into the competitive dynamics and partial cross-immunity between these two pathogens. The refined and validated model, grounded in real-world data, seeks to enhance our understanding of pathogen interactions, inform targeted public health interventions, and offer a clear and replicable framework for future research, ultimately aiding in the effective management of cocirculating respiratory viruses.

## Methods

### Data

We used officially reported data on SARS-CoV-2 and influenza infections from the Robert Koch Institute, Germany’s national public health authority [44]. Age-specific population data were sourced from the Federal Statistics Office of Germany [45]. The reported cases span from the first week of 2020 to the last week of 2022, a period that captures key developments in the COVID-19 pandemic, including vaccine rollouts, and allows assessment of interactions between SARS-CoV-2 and influenza, including potential cross-immunity effects [46].

Data include weekly age-stratified reports of new infections, summarized in Table 1. This age-based classification enables targeted epidemiological analysis in critical life stages, supporting effective public health planning.

**Table 1:** Cumulative new cases stratified by age groups

	Age of participants ($a_j$)	Sum of the number of participants (New cases)		Total
*j*		Influenza data	Covid-19 data	
1	0-4 years	44348	1015561	1059909
2	5-14 years	95847	4471306	4567153
3	15-34 years	114777	10940785	11055562
4	35-59 years	117369	14550028	14667397
5	60-79 years	52056	4896335	4948391
6	80+ years	25635	1509503	1535138
	**Total**	441532	37283918	37725450

**Fig. 1:**
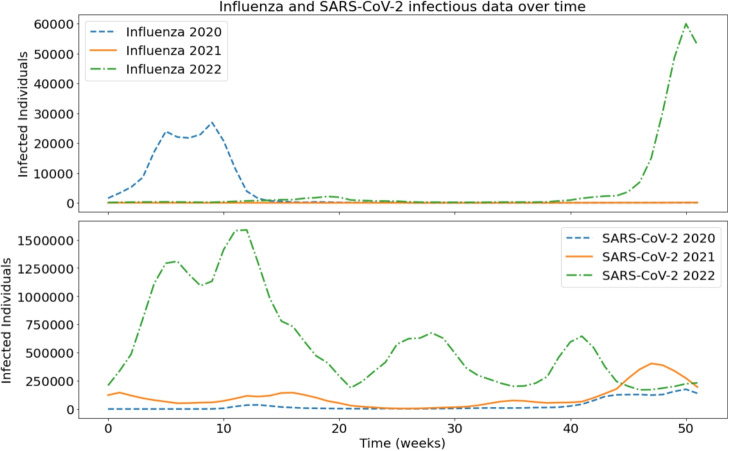
Weekly number of infected individuals for Influenza (top) and SARS-CoV-2 (bottom) during the years 2020, 2021, and 2022. The plots show the temporal evolution of reported infections, highlighting the seasonal pattern of Influenza and the multiple waves of SARS-CoV-2. While Influenza infections peaked only in 2020, SARS-CoV-2 demonstrated sustained high transmission across all three years, with a notably sharp increase in 2022

Figure 1 illustrates the weekly evolution of the infections reported for both diseases from 2020 to 2022. Influenza cases show a distinct seasonal pattern, with a sharp peak in early 2020 (weeks 10- 12), followed by a rapid decline likely influenced by containment measures such as lockdowns and non-pharmaceutical interventions (NPIs). Influenza activity remained extremely low in 2021 and 2022, according to global observations, mainly due to mask use, travel restrictions, school closures, and increased awareness of hygiene. In contrast, SARS-CoV-2 displayed multiple waves throughout the 3-year span. In 2020, moderate case numbers decreased under early containment strategies. In 2021, peaks appeared around weeks 45–50, corresponding to the Delta variant wave. The most significant surge occurred in 2022, with cases exceeding 1.5 million per week during the Omicron wave (around week 12).

In general, this period captures a significant epidemiological shift: from seasonal influenza prevalence in early 2020 to the dominant and persistent burden of SARS-CoV-2 thereafter. The data set provides a unique opportunity to examine the interaction between these viruses under rapidly evolving public health conditions.

### Model description and social contact patterns

The model used in this study is adapted from the extended SEIQR framework developed in our previous work [37]. In this study, we further expand the model by incorporating age-structured heterogeneity to capture pathogen dynamics across six age groups. As highlighted in Section 1, we exclude co-infection compartments due to their low prevalence and focus on the interaction between individual pathogens and cross-immunity effects.

The population is assumed to be well mixed with equal contact opportunities. A constant birth rate ($\Lambda$) replenishes the susceptible class, while natural deaths ($\mu$) and rare disease-induced deaths ($\delta _i, \delta _c$) stabilize the total population size over time [47]. Disease transmission occurs from infectious individuals ($I_i, I_c$) to susceptible individuals at rates $\beta _i$ and $\beta _c$, resulting in exposed classes ($E_i, E_c$), which transition to infectious states via rates $\rho _i$ and $\rho _c$. The quarantine and isolation measures are imperfect; Transitions from exposed and infectious compartments to quarantine ($Q$) and isolation occur at rates $\alpha$ and $\sigma$, respectively, and quarantined individuals can progress to infectious states at a leakage rate $\tau$. Recovery occurs at rates $\gamma$ and $\phi$.

Recovered individuals can acquire secondary infections due to partial cross-immunity, governed by $\eta ^c_i$ and $\eta ^i_c$, where the value 0 denotes complete protection and the value 1 indicates no protection. These parameters were derived from [48], which provides key insights into how immune responses influence the dynamics of epidemics. The study estimates that infection rates range from 0% to 100%, offering a foundation for understanding the impact of antigenic variation on infection rates and immunity. To incorporate “partial and complete cross-immunity” into the model, as shown in Fig. 9, we modify the transmission probabilities based on previous immunity to the first pathogen. For complete cross-immunity, it is assumed that individuals immune to the first pathogen have complete protection against the second, which is represented by setting $\eta ^{c}_{i}$ and $\eta ^{i}_{c}$ to zero, indicating no susceptibility. For partial cross-immunity, a reduction factor (ranging 0 and 1) is applied to the transmission probability, reflecting reduced susceptibility but not full immunity.

To account for the age structure, as detailed in Table 1, we stratify all compartments into six age-specific sub-compartments, resulting in a total of 54 compartments. The total population at time $t$ is:

1 $$\begin{aligned} N_T(t) = \sum \limits _{j=1}^{6}S^{a_j}(t) + \sum \limits ^6_{{j=1},x \in \{i, c\}} (E^{a_j}_x(t) + I^{a_j}_x(t) + Q^{a_j}_x(t) + R^{a_j}_x(t)). \end{aligned}$$

The ordinary differential equation (ODE) system for the age group $j=1$ is presented in Eq. (2). For the remaining age groups ($j = 2, \dots , 6$), the structure is identical, except that the susceptible compartment does not receive birth inflows. Due to the short duration of the disease cycles, migration between age groups is not considered. The corresponding compartmental transitions for the age group $j=1$ are illustrated in Fig. A1 in the Appendix, and all model parameters are listed in Table 2.

2 $$\begin{aligned} S'^{a_j}(t)= & \Lambda - \xi _{i}^{a_{j}}(t)S^{a_j}(t)-\xi _{c}^{a_{j}}(t)S^{a_j}(t) - \mu S^{a_j}(t), \nonumber \\ E'^{a_j}_{i}(t)= & \xi _{i}^{a_{j}}(t)S^{a_j}(t)+ \eta ^{i}_cR_c^{a_j}(t)\xi _{i}^{a_{j}}(t) - (\alpha _i +\rho _i +\mu )E_{i}^{a_j}(t),\nonumber \\ E'^{a_j}_{c}(t)= & \xi _{c}^{a_{j}}(t)S^{a_j}(t)+ \eta ^{c}_iR_i^{a_j}(t)\xi _{c}^{a_{j}}(t)- (\alpha _c +\rho _c +\mu )E_c^{a_j}(t),\nonumber \\ I'^{a_j}_i(t)= & \rho _iE_i^{a_j}(t)+\tau _iQ_i^{a_j}(t)-(\gamma _{i}+\sigma _{i}+\delta _{i}+\mu )I_i^{a_j}(t),\nonumber \\ I'^{a_j}_c(t)= & \rho _cE_c^{a_j}(t)+\tau _cQ_c^{a_j}(t) - (\gamma _{c}+\sigma _{c}+\delta _{c}+\mu )I_c^{a_j}(t),\nonumber \\ Q'^{a_j}_i(t)= & \alpha _iE_i^{a_j}(t)-(\phi _{i}+\tau _{i}+\delta _{i}+\mu )Q_i^{a_j}(t)+\sigma _iI_i^{a_j}(t),\nonumber \\ Q'^{a_j}_c(t)= & \alpha _cE_c^{a_j}(t)-(\phi _{c}+\tau _{c}+\delta _{c}+\mu )Q_c^{a_j}(t)+\sigma _cI_c^{a_j}(t),\nonumber \\ R'^{a_j}_{i}(t)= & \phi _iQ_i^{a_j}(t)+\gamma _iI_i^{a_j}(t)-\eta ^{c}_iR_i^{a_j}(t)\xi _{c}^{a_{j}}(t)-\mu R_i^{a_j}(t),\nonumber \\ R'^{a_j}_{c}(t)= & \phi _cQ_c^{a_j}(t)+\gamma _cI_c^{a_j}(t)-\eta ^{i}_cR_c^{a_j}(t)\xi _{i}^{a_{j}}(t)-\mu R_c^{a_j}(t). \end{aligned}$$

From Eq. 2, the vectors $\xi _{c}(t)$ and $\xi _{i}(t)$ represent the force of infection rates for COVID-19 and influenza, respectively, across six age groups. For a specific age group $a_{j}$, they are given by:

3 $$\begin{aligned} \xi _{i}^{a_{j}}(t)= & \sum \limits _{l=1}^{6}\chi ^{a_{jl}}_i\beta ^{a_j}_i(t)\left[ I_i^{a_j}(t)+\Omega _{i}Q_i^{a_j}(t)\right] ,\nonumber \\ \xi _{c}^{a_{j}}(t)= & \sum \limits _{l=1}^{6}\chi ^{a_{jl}}_c\beta ^{a_j}_c(t)\left[ I_c^{a_j}(t) +\Omega _{c}Q_c^{a_j}(t)\right] . \end{aligned}$$

**Table 2:** Descriptions, values, and data sources of model parameters

Symbol	Definition	Values	Unit	Source
*c*	Contact rate	$[0, 1]^{}$	day$^{-1}$	[27]
$\Lambda$	Birth rate	(0,0.000024]	day$^{-1}$	this study
$\mu$	Natural death rate	(0,0.000024]	day$^{-1}$	this study
$\beta _c$	COVID-19 transmission probabilities per contact	[0, 1]	day$^{-1}$	this study
$\beta _i$	Influenza transmission probabilities per contact	[0, 1]	day$^{-1}$	this study
$\rho _c$	Transition rate from $E_c$ to $I_c$	[1/4.3, 1/2.3]	day$^{-1}$	[27]
$\rho _i$	Transition rate from $E_i$ to $I_i$	[1/5, 1/2]	day$^{-1}$	[49]
$\alpha _{c}$	$E_c$ quarantine rate	[0.89, 1.51]	day$^{-1}$	[50]
$\alpha _{i}$	$E_i$ quarantine rate	[0.89, 1.51]	day$^{-1}$	[50]
$\sigma _{c}$	$I_c$ isolation rate	[0.02, 0.1]	day$^{-1}$	[49]
$\sigma _{i}$	$I_i$ isolation rate	[0.02, 0.1]	day$^{-1}$	[17]
$\tau _{c}$	Progression rate from $Q_c$ to $I_{c}$	(0, 0.5]	day$^{-1}$	[51]
$\tau _{i}$	Progression rate from $Q_i$ to $I_{i}$	(0, 0.5]	day$^{-1}$	[51]
$\phi _{c}$	$Q_c$ recovery rate	[0.08, 0.14]	day$^{-1}$	[50]
$\phi _{i}$	$Q_i$ recovery rate	[0.08, 0.14]	day$^{-1}$	[50]
$\gamma _{c}$	$I_c$ recovery rate	[0.28, 0.38]	day$^{-1}$	[50]
$\gamma _{i}$	$I_i$ recovery rate	[1/6, 1/4]	day$^{-1}$	[52]
$\delta _{c}$	Death rate due to COVID-19	(0, 1)	day$^{-1}$	[37]
$\delta _{i}$	Death rate due to influenza	(0, 1)	day$^{-1}$	[49]
$\eta ^i_{c}$	Cross-immunity against COVID-19	[0, 1]	-	[48]
$\eta ^c_{i}$	Cross-immunity against influenza	[0, 1]	-	[48]

The square bracketed terms represent individuals who can transmit the virus. The parameters $\Omega _{i}$ and $\Omega _{c}$ (with values in $(0,1]$) account for the imperfect quarantine. These factors quantify the effectiveness of quarantine in reducing, although not completely preventing, the risk of transmission, focusing on the role of education and awareness of infection dynamics [53]. The time-dependent probabilities $\beta ^{a_j}_i(t)$ and $\beta ^{a_j}_c(t)$ reflect the transmission rates per contact for influenza and SARS-CoV-2, respectively. The coefficients $\chi _k^{a_{jl}} = M_{a_{jl}} / N^{a_j}$ normalize age-specific contact rates by population size, where $k \in \{i, c\}$ and $M_{a_{jl}}$ represent average daily contacts from POLYMOD data. For complete details of the social contact matrix and its derivation, see the Appendix.

### Age-specific quarantine reproduction number

To assess the potential for disease spread under control measures, we evaluate the age-specific quarantine reproduction numbers for COVID-19 and influenza using the next generation matrix approach. The detailed derivation, including the identification of infected compartments, formation of the next generation matrix, and derivation of expressions for each age group, is provided in Appendix. The age-specific quarantine reproduction numbers for COVID-19 $\tilde{\mathcal {R}}^{a_j}_c$ and influenza $\tilde{\mathcal {R}}^{a_j}_i$ quantify the expected number of secondary infections in age group $a_j$, accounting for control measures such as quarantine. We define the overall control reproduction number as:

$$\begin{aligned} \tilde{\mathcal {R}}^q = \max \left\{\tilde{\mathcal {R}}^{a_j}_c, \tilde{\mathcal {R}}^{a_j}_i \text { for all } j\right\}. \end{aligned}$$

This provides a measure of the worst-case potential for disease spread under current control measures between different age groups. If $\tilde{\mathcal {R}}^q$ exceeds 1, it indicates that despite the control measures, the disease could continue to spread. In contrast, if it is less than 1, it suggests that the control measures are effective in reducing the spread of the diseases below the threshold where an outbreak could be sustained.

### Sensitivity analysis

A key methodological approach in modeling that assists in determining the most important parameters that influence model results is global sensitivity analysis (GSA) [54, 55]. To evaluate the uncertainty between model inputs and outputs in epidemic modeling, GSA techniques have been used in a number of studies [56–58]. To capture the effects of individual parameters and their interactions, for example, [56] used Pearson correlation coefficients, where-as [57, 58] used the Sobol sensitivity technique, a variance-based GSA method.

In this section, we apply the Sobol sensitivity technique to the age-unspecific model, concentrating on the important parameters listed in Table 2. Through a methodical manipulation of these variables over their potential domains, GSA evaluates the resilience of our model’s forecasts and pinpoints the most consequential variables. Furthermore, GSA sheds light on parameter interactions by providing a thorough understanding of how many adjustments might impact the behavior of the model [54, 55, 59]. Recognizing the sensitivity of partial cross-immunity between COVID-19 and influenza parameters can aid in forecasting disease trends and developing management plans. We have included a detailed study of our methods in Appendix.

### Model fitting

The initial stage of data fit involved managing missing values and rectifying custom inconsistencies for each pathogen-specific dataset, thus ensuring data integrity. Histograms were used to visualize the distributions, offering insight into the spread and helping to identify notable patterns or outliers. To normalize the data and reduce the impact of skewness or outliers, transformation techniques were applied, including logarithmic, square root, Box-Cox, and power transformations. Following data transformation, the compartmental model was initialized with parameter sets sampled from the same predefined ranges used in the Sobol sensitivity analysis and then fitted to the normalized data (such as age-specific total population) using the least squares optimization method. We utilized the ’scipy.optimize.least_squares’ function with bounded constraints to ensure biological plausibility of parameter estimates [60]. For multi-pathogen models (including both unspecified and age-specified variants), where transformed variables did not satisfy normality, a nonparametric approach was employed. Specifically, 10,000 bootstrapped samples were generated to estimate medians and confidence intervals, following the methodology described by [61].

To ensure that the model output aligned with the structure of the empirical data, we carefully considered the nature of the observational data. In fact, the variables $I_i(t)$ and $I_c(t)$ in our model represent the number of currently infectious individuals at time $t$, corresponding to prevalence. However, the empirical data used for the fitting of the model consist of new cases reported weekly, representing the incidence. To address this mismatch appropriately, we did not fit the prevalence variables $I_i(t)$ and $I_c(t)$ directly to the incidence data. Instead, we derived the weekly incidence based on the model by calculating the rate of new infections entering the exposed compartments $E_i(t)$ and $E_c(t)$, using the infection terms in the model. This allowed for a consistent comparison between model output and reported data.

Importantly, different initial conditions were assigned to reflect the different epidemiological contexts of SARS-CoV-2 and influenza at the beginning of 2020. For SARS-CoV-2, we assumed a completely susceptible population without prior immunity ($R_0 = 0$). In contrast, for influenza, the model included a non-zero initial recovered state to reflect pre-existing immunity. To estimate the number of individuals recovering from influenza, we used the total population of Germany in early 2020 (N = 83,166,711) and distributed it in six age groups based on demographic data of the population. The age-specific influenza attack rates for the 2012/13 season, as reported by the Robert Koch Institute [62] were then applied to each age group to estimate the total number of individuals with prior infection. The resulting recovered counts were: 00–04 years $\approx$ 586,477; 05-14 years $\approx$ 1,529,469; 15-34 1 years $\approx$ 896,102; 35-59 years $\approx$ 2,328,668; 60–79 years $\approx$ 1,062,531; and 80+ years $\approx$ 282,567. These estimates were normalized by the total population to obtain the initial proportions recovered for each age group. The obtained values were used to initialize the recovered compartment in the influenza model, thereby capturing the pre-existing immunity structure in the population. Finally, statistical tests, such as the t test or the Mann-Whitney U test, were used to assess whether the estimated parameters differed significantly between the groups, with a significance threshold of 0.05.

## Results

### Model fit

**Fig. 2:**
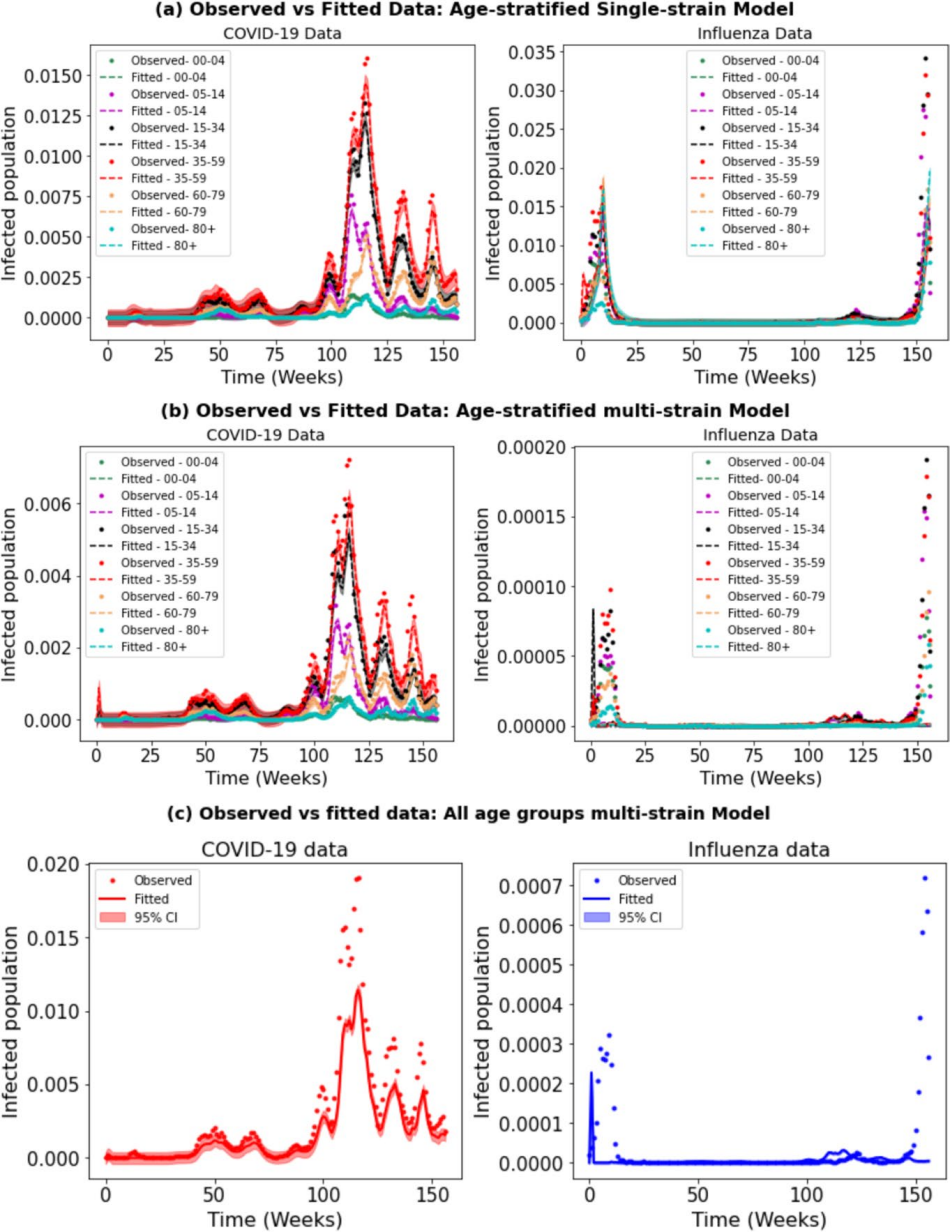
Assessing the adequacy of the SARS-CoV-2 and Influenza models by comparing their fit to observed data. The bandwidth illustrates the 95% confidence intervals for the fitted data. On the X-axis, ‘0’ corresponds to the first week of 2020, while ‘157’ indicates the final week of 2022

**Fig. 3:**
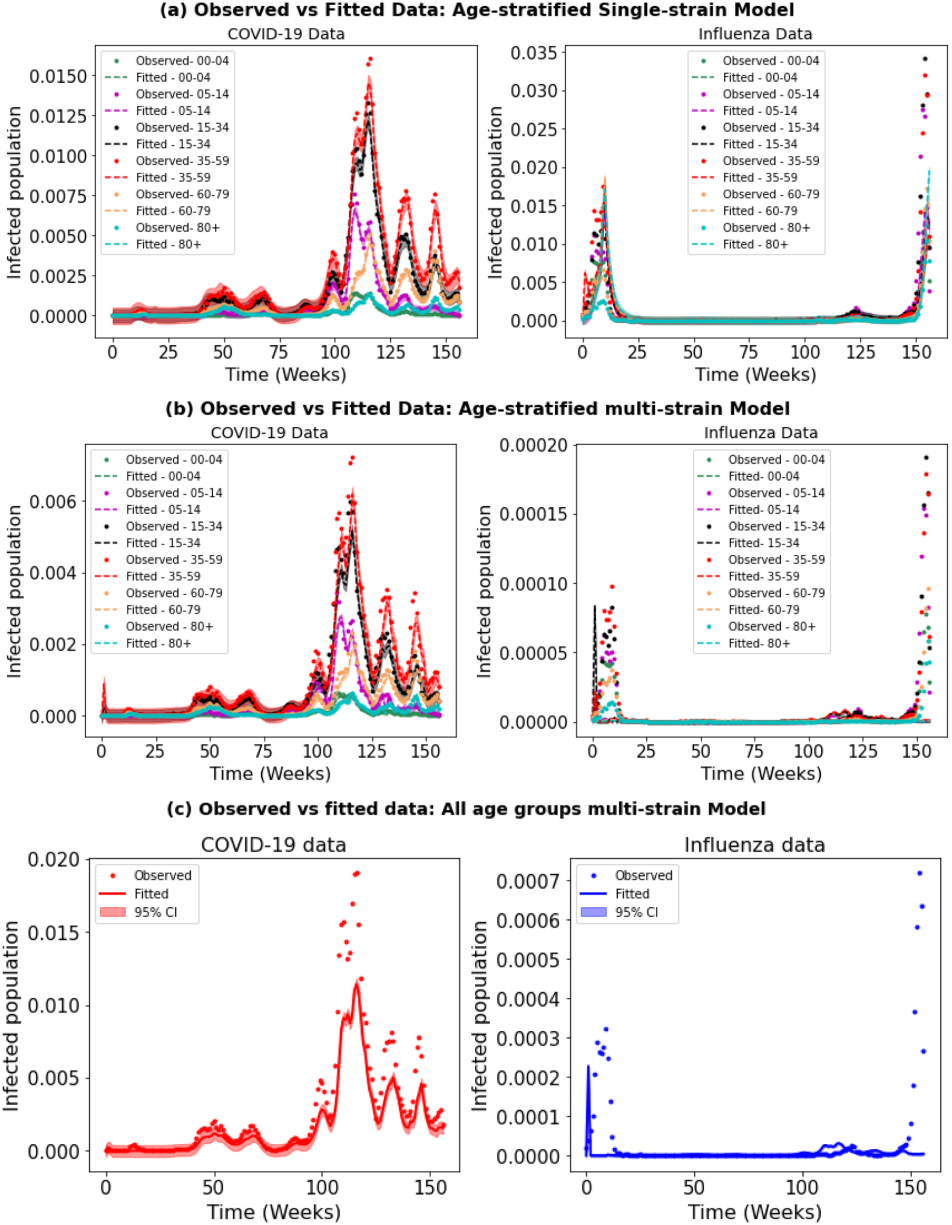
Assessing the adequacy of the SARS-CoV-2 and Influenza models by comparing their fit to observed data, fitted to the normalized data (such as age-specific total population). The bandwidth illustrates the 95% confidence intervals for the fitted data. On the X-axis, ‘0’ corresponds to the first week of 2020, while ‘157’ indicates the final week of 2022

We fitted our model to real-world data using parameter sets from sensitivity analysis. This method includes both single- and dual-pathogen models, using age-specific parameters to account for differences in how diseases spread and affect various age groups. The estimated parameters for single-pathogen models help us to understand how each pathogen spreads on its own. This is important for accurately expanding the model to handle interactions between two pathogens, such as competition or co-existence (Fig. 2). For example, in subplot (a) of Fig. 3, each pathogen is modeled separately, and the fit closely matches the observed data. In contrast, in subplot (b), where both pathogens are modeled together, influenza peaks early but then decreases, while COVID-19 becomes more dominant. Although influenza increases slightly toward the end, its numbers remain lower than COVID-19, showing that influenza has been overtaken. Furthermore, compared to a model that combines all age groups, our age-specific model shows improved precision in fitting the data, as demonstrated in the Fig. subplot 3c.

### Estimated transmission rates

**Fig. 4:**
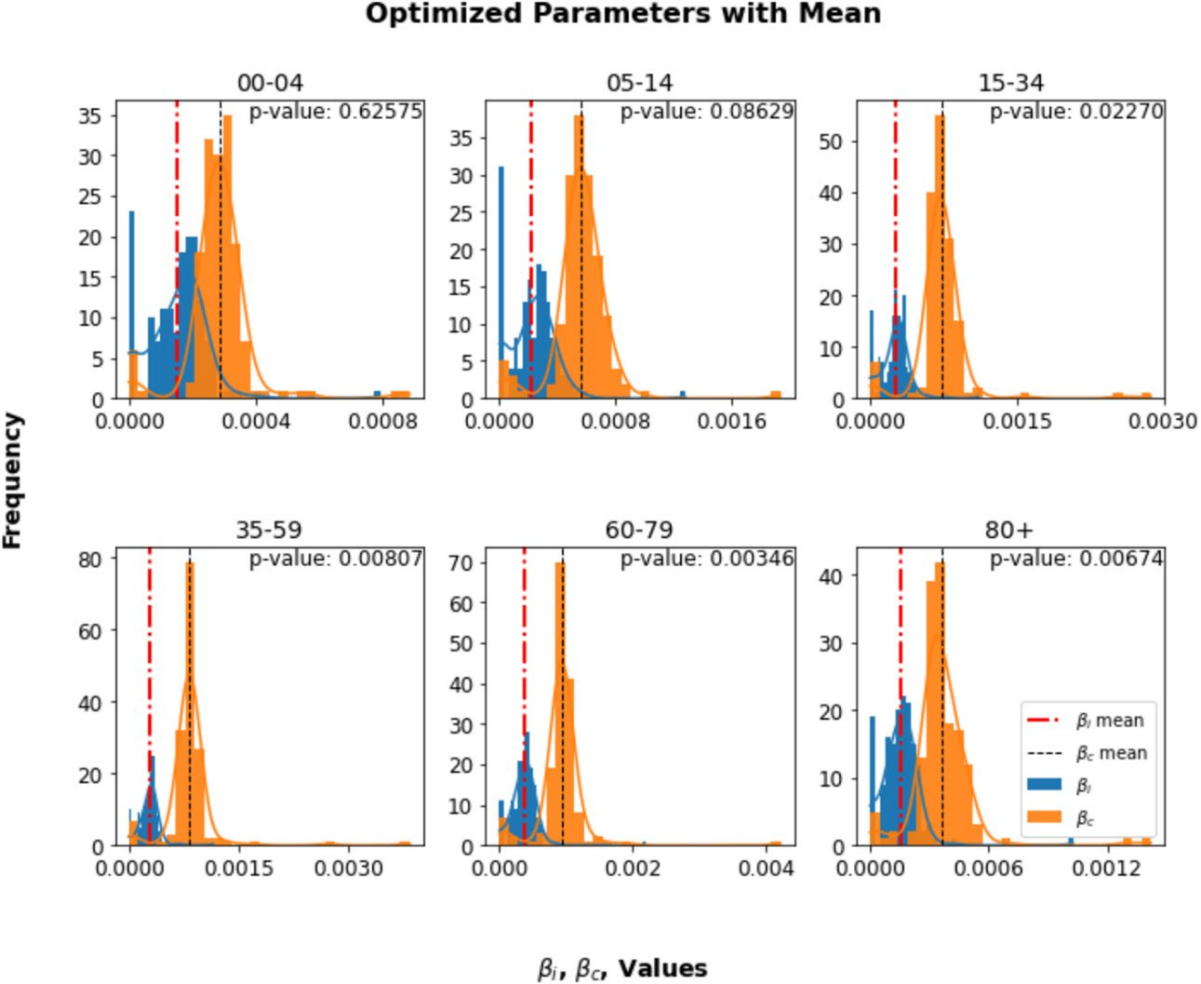
Histograms with kernel density estimates (KDE) illustrating the optimized transmission parameters ($\beta _i$ and $\beta _c$) in the single-pathogen model, along with their mean values and a statistical comparison using a t-test. Blue histograms represent $\beta _i$, and orange histograms represent $\beta _c$. Dashed lines indicate the mean values, and the *p*-value of the t-test is shown. The x-axis represents parameter values, and the y-axis shows the frequency of those values

**Fig. 5:**
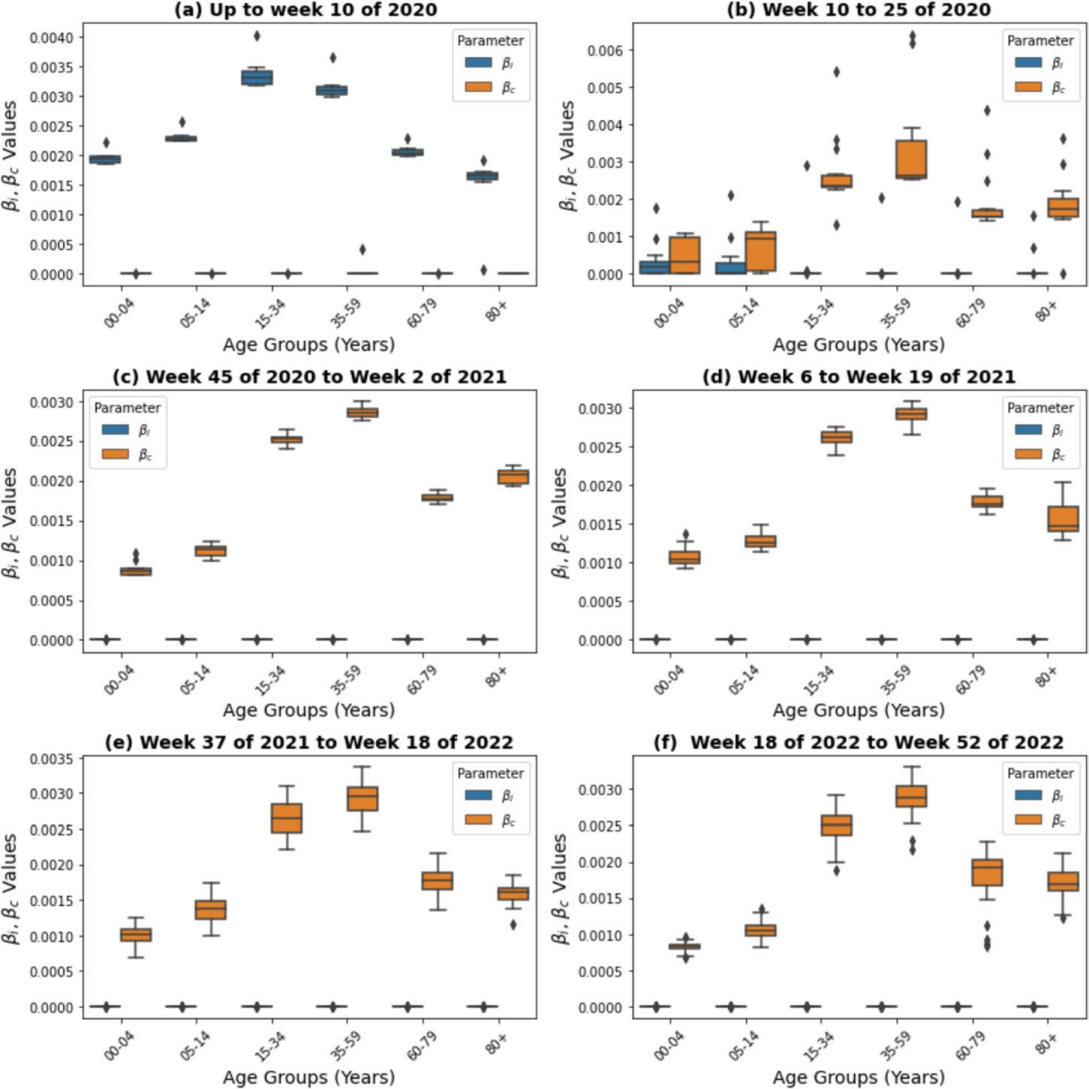
Box-plots of $\beta _{i}$ and $\beta _{c}$ values across age groups in a multi-pathogen model for each pandemic wave. Each subplot represents a time interval from the first (**a**) to the sixth and seventh waves (**f**). The central line in each box is the median, the box represents the interquartile range (IQR), and whiskers extend to 1.5 times the IQR from the box edges, with outliers shown as points

Table 3 presents the transmission probability parameters ($\beta _i$ and $\beta _c$) estimated for both the single-pathogen and multi-pathogen models. The time-dependent transmission probabilities, including those for the multi-pathogen model, are presented in Fig. 8, where the temporal dynamics are explicitly considered. The distributions of these parameters across various time periods and age groups are depicted in Figs. 4 and 5. Specifically, Fig. 4 illustrates the distribution of transmission parameters ($\beta _i$ and $\beta _c$) for the single pathogen model in different age groups, showing variations within each group. The mean values are highlighted, and the *p*-values of the t test indicate the significance of the differences between the pathogens, providing information on how the transmission dynamics vary between pathogens and among demographics of age. The findings illustrated in Fig. 5 shed light on the dynamics of the estimated infection probability rates for both influenza ($\beta _i$) and COVID-19 ($\beta _c$) in the multi-pathogen model over a specified time frame. In subplot (a), during the initial phase of the simulation until week 10 of 2020, the median influenza transmission rates ($\beta _i$) remain relatively consistent between age groups, ranging from approximately $1.7 \times 10^{-3}$ to $3.2 \times 10^{-3}$. These values suggest a moderate to high probability of influenza infections in all age groups, with slightly higher transmission rates observed in the age groups 15-34 and 35-59 compared to others. In contrast, the median transmission rates for COVID-19 ($\beta _c$) are considerably lower between all age groups during this period, ranging from approximately $1.7 \times 10^{-16}$ to $7.4 \times 10^{-13}$. These values indicate a very low probability of COVID-19 infections, with transmission rates nearly negligible, especially in the 00-04 and 05-14 age groups. This aligns with the observed cases, as these age groups exhibit significantly lower cumulative new cases of COVID-19 compared to other age groups such as 15-34 and 35-59, supporting the notion of minimal transmission in these cohorts during the period under review.

**Table 3:** The estimated parameters include mean and standard error values for $\beta _i$ and $\beta _c$ in the single-pathogen model, and median values with 95% confidence intervals (lower bound, upper bound) for the multi-pathogen model. We used the mean and standard error for the single-pathogen model because the estimates were relatively stable across runs. In contrast, the multi-pathogen model showed greater variability due to pathogen interactions; thus, we reported the median and CI to better reflect the central tendency and uncertainty

**Single-pathogen model**			
**Age (years)**	$\beta _i$**Mean(SE)**	$\beta _c$**Mean(SE)**	***P*-values**
0-4	1.5$\times 10^{-4}$(7.8$\times 10^{-6}$)	2.8$\times 10^{-4}$(8.2$\times 10^{-6}$)	0.6257
5-14	2.2$\times 10^{-4}$(1.3$\times 10^{-5}$)	5.6$\times 10^{-4}$(1.5$\times 10^{-5}$)	0.0862
15-34	2.5$\times 10^{-4}$(1.1$\times 10^{-5}$)	7.3$\times 10^{-4}$(2.3$\times 10^{-5}$)	0.0227
35-59	2.8$\times 10^{-4}$(1.0$\times 10^{-5}$)	8.3$\times 10^{-4}$(2.8$\times 10^{-5}$)	0.0080
60-79	3.7$\times 10^{-4}$(1.7$\times 10^{-5}$)	9.3$\times 10^{-4}$(2.9$\times 10^{-5}$)	0.0034
80+	1.5$\times 10^{-4}$(8.3$\times 10^{-6}$)	3.6$\times 10^{-4}$(1.2$\times 10^{-5}$)	0.0067
**Multi-pathogen model**			
**Age (years)**	$\beta _i$**Median (CI)**	$\beta _c$**Median (CI)**	***P*-values**
0-4	6.2$\times 10^{-12}$(2.3$\times 10^{-12}$,1.5$\times 10^{-11}$)	0.0009(8.7$\times 10^{-4}$,9.7$\times 10^{-4}$)	$<0.0001$
5-14	1.9$\times 10^{-13}$(2.7$\times 10^{-14}$,4.8$\times 10^{-13}$)	0.0014(1.2$\times 10^{-3}$,1.5$\times 10^{-3}$)	$<0.0001$
15-34	3.5$\times 10^{-15}$(1.4$\times 10^{-16}$,1.8$\times 10^{-14}$)	0.0024(2.3$\times 10^{-3}$,2.5$\times 10^{-3}$)	$<0.0001$
35-59	2.7$\times 10^{-15}$(1.5$\times 10^{-16}$,1.2$\times 10^{-14}$)	0.0028(2.7$\times 10^{-3}$,2.9$\times 10^{-3}$)	$<0.0001$
60-79	1.2$\times 10^{-13}$(1.4$\times 10^{-14}$,7.3$\times 10^{-13}$)	0.0024(2.3$\times 10^{-3}$,2.5$\times 10^{-3}$)	$<0.0001$
80+	1.0$\times 10^{-13}$(5.2$\times 10^{-15}$,4.5$\times 10^{-13}$)	0.0012(1.1$\times 10^{-3}$,1.2$\times 10^{-3}$)	$<0.0001$

From week 10 to 25 of 2020 (as described in Fig. 5 subplot b), the older age groups (05-14, 15-34, 35-59, 60-79, 80+) exhibit lower median flu infection probability rates compared to the youngest age group, 00-04. The age group 00-04 shows a median transmission rate of approximately $0.0001671$, with a confidence interval ranging from $8.33 \times 10^{-8}$ to $0.0003237$. In contrast, for COVID-19 transmission, the 00-04 age group now shows the lowest median transmission rate among all age groups, approximately $0.0003176$. However, similar to influenza, the wide confidence interval indicates considerable uncertainty regarding the actual transmission rate within this age group. This uncertainty may arise from factors such as limited data availability, variations in test rates, and differences in exposure risks. Furthermore, social contact patterns play a crucial role; during the pandemic, younger children, particularly those aged 0 to 4, may have had more frequent contacts within home settings due to daycare closures and parental supervision during remote work periods, potentially influencing transmission dynamics [63]. Studies have also shown that children, especially those under 10 years of age, have different susceptibility and transmission dynamics compared to adults, which could contribute to the observed lower transmission rates and associated uncertainties in this age group [64].

Generally, the median values for COVID-19 ($\beta _c$) show an increase compared to the previous period, ranging from approximately $0.3 \times 10^{-3}$ to $1.7 \times 10^{-3}$ across different age groups. Data for week 45 of 2020 to week 52 of 2022 (as shown in subplots (c) to (f) of Fig. 5) underscore the increased prevalence of COVID-19 compared to influenza, and COVID-19 exhibits higher median transmission probability values across most age groups. This suggests a higher probability of COVID-19 infections during this period, particularly in the 15–34 and 35–59 age groups, where the highest $\beta _c$ values are observed. However, estimates for COVID-19 transmission show wider interquartile ranges (IQRs) compared to influenza, indicating greater variability and uncertainty in COVID-19 transmission estimates. This is likely due to greater heterogeneity in cases or dynamic interventions during the pandemic [65].

The epidemiological plausibility of these estimated transmission probabilities ($\beta _i$ and $\beta _c$) was assessed indirectly by analyzing the resulting age-specific reproduction numbers ($\tilde{\mathcal {R}}_i^{a_j}$ and $\tilde{\mathcal {R}}_c^{a_j}$), presented in the following section. The estimated secondary cases in age groups and over time aligned with the known transmission dynamics of influenza and COVID-19, supporting the realism of the estimated parameters $\beta$.

### Estimated number of secondary cases

**Fig. 6:**
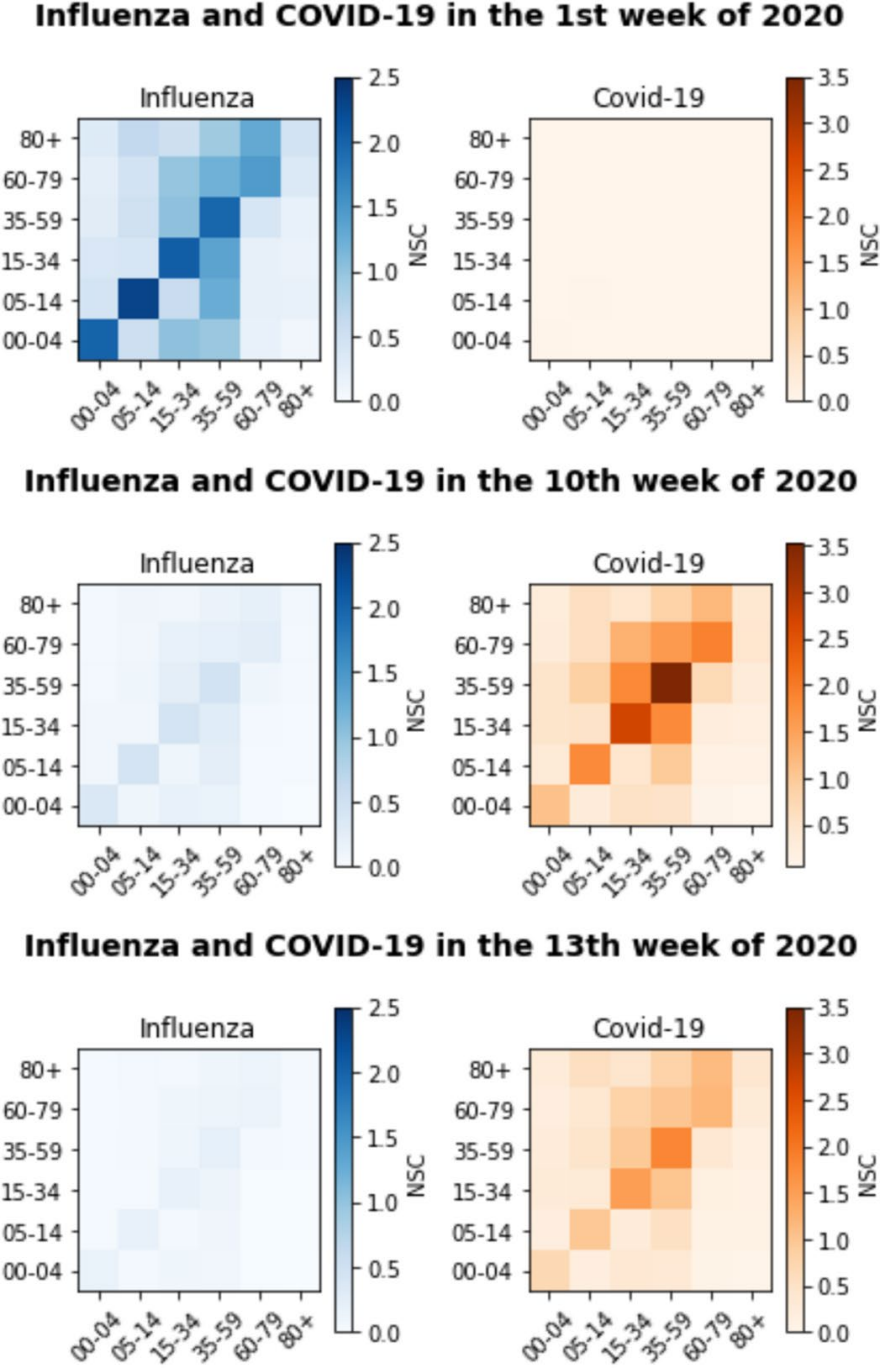
Quarantine reproduction number for both influenza and COVID-19 across different age groups during the initial COVID-19 wave at selected weeks. The x-axis represents the age of individuals (years), while the y-axis represents the age of secondary cases (years). NSC: Number of secondary cases

**Fig. 7:**
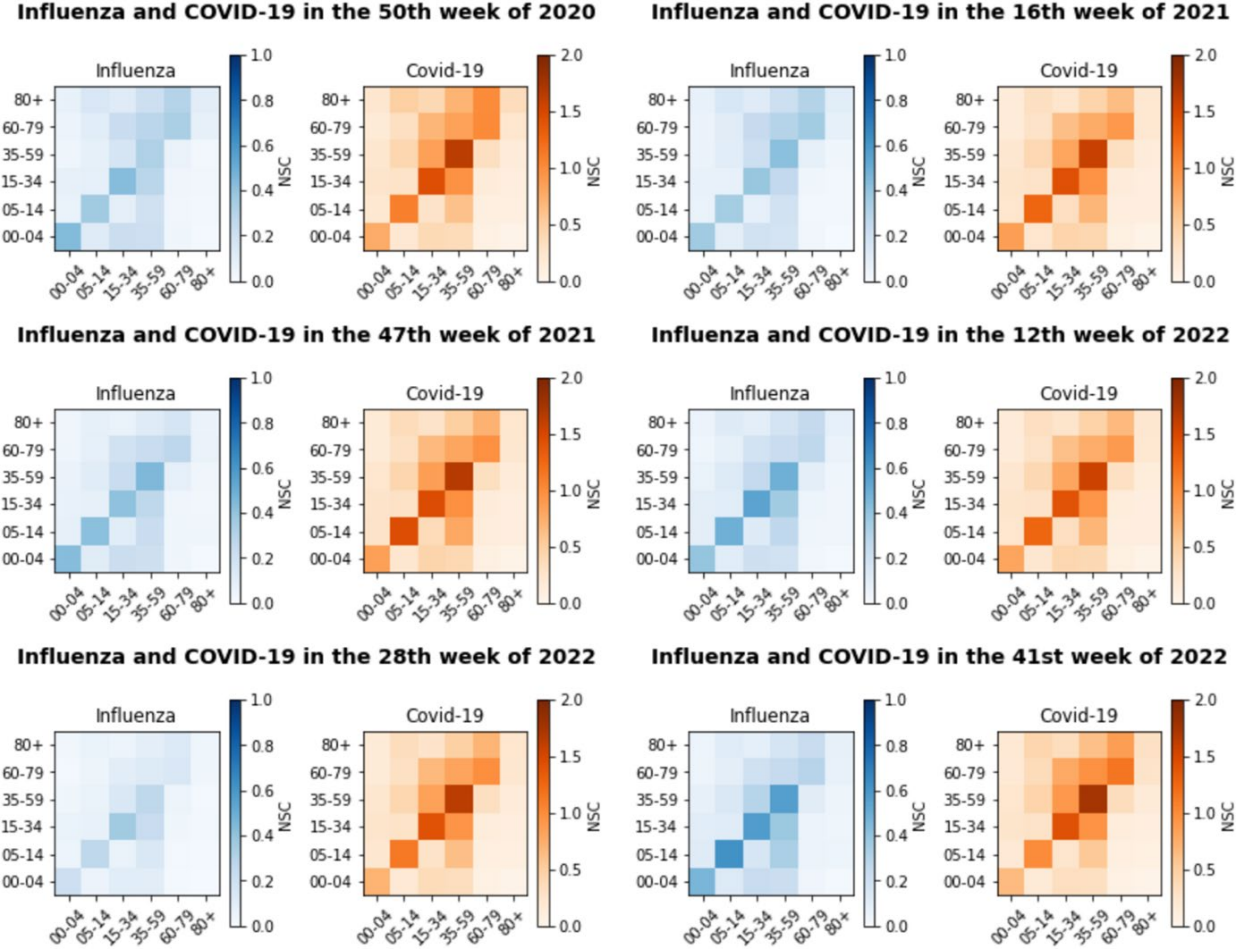
Estimates of quarantine reproduction number for influenza and COVID-19 across age groups during peak weeks from the second to seventh waves. X-axis: Age of individuals (years), Y-axis: Age of secondary cases (years). NSC: Number of secondary cases

Utilizing Equation C3 in the appendix, estimations of secondary cases associated with specific age groups for each pathogen are provided weekly (see Figs. 6 and 7). These results highlight the different transmission dynamics of influenza and COVID-19 between age groups. In particular, during the first week of 2020, no cases of COVID-19 were reported, consistent with the absence of secondary cases recorded. In contrast, influenza exhibited its highest estimated quarantine reproduction number in the 05-14 age group at 2.31, suggesting a potential transmission to approximately 2.31 individuals within the same age group per infected individual. Subsequent transmission rates for other age groups that interact with individuals in the same age group were: 15-34 (2.05), 00-04 (2.00), 35-59 (1.98), 60-79 (1.45) and 80 + (0.65). In Week 10, the estimated number of influenza quarantine reproduction in various age groups was significantly lower, with values ranging from 0.01 to 0.46. In contrast, for COVID-19, the highest estimated number of quarantine reproduction was observed in the 35-59 age group (3.53), followed by the 15-34 age group (2.72), the 60-79 age group (1.92), the 05-14 age group (1.80), the 80 + age group (0.42) and the 00-04 age group (0.30). In Week 13, the estimated number of influenza quarantine reproduction remained consistently low, below 0.2 for most age groups. However, for COVID-19, there was a decrease compared to the previous week, with values ranging from 0.12 to 1.82 in different age groups.

In subsequent selected weeks, ranging from Week 50 of 2020 through Week 41 of 2022—which mark the peak periods of successive pandemic waves—patterns similar to those in Fig. 6 are observed. Throughout these weeks, the highest reproduction number appears consistently in the 35-59 age group, indicating a greater transmission potential within this demographic. Meanwhile, the variation in age-specific reproduction numbers in other age groups, as shown in Fig. 7, reflects the changes in effective contact patterns and susceptibility over time. These changes highlight the dynamic nature of inter-age group interactions during different phases of the pandemic. During this period (Week 50 of 2020 to Week 41 of 2022), the dynamics of influenza transmission shows the highest values in the age group 05-14, followed by the age groups 35-59 and 60-79, with their values consistently remaining below 1 in different age groups. This suggests that while some transmission occurs, it remains relatively low in most demographics during this time frame (see Fig. 7).

### Dominance and extinction of pathogens

**Fig. 8:**
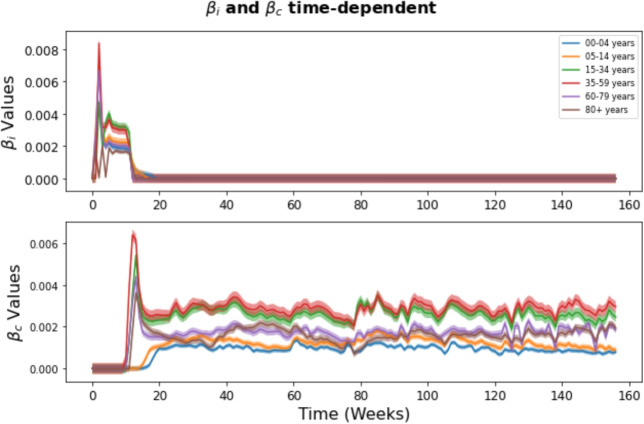
Time-dependent estimates of the parameters $\beta _i$ and $\beta _c$ in the multi-pathogen model. The X-axis represents time, with ‘0’ marking the first week of 2020 and ‘157’ indicating the final week of 2022. The shaded areas represent the 95% confidence intervals for the parameter estimates

Figure 8 presents the time-varying infection rates ($\beta _i$ for influenza and $\beta _c$ for COVID-19), along with their 95% confidence intervals (CI), for each age group, as estimated from the multi-pathogen model. These trajectories offer insight into how transmission patterns evolved over time while accounting for uncertainty in the estimation. The figure reveals a clear shift: influenza transmission initially dominated in early 2020, but was rapidly overtaken by COVID-19. Influenza activity remained low for the remainder of the study period, while COVID-19 transmission persisted with variable intensity. This trend reflects the dynamic interaction between the two viruses, highlighting the need for continuous surveillance and adaptive public health strategies to effectively respond to overlapping respiratory outbreaks.

### Sensitivity analysis outcomes

Sobol sensitivity indices are used to quantify how variations in the model parameters influence the output. The first-order Sobol index ${\textbf {SI}}_{st}^{P_{i}}$ estimates the direct effect of a single parameter $P_i$ on output variability, while the total-order Sobol index ${\textbf {SI}}_{tot}^{P_{i}}$ accounts for both direct effects and all higher-order interactions that involve $P_i$.

Table 4 highlights $\beta _i$ and $\beta _c$ as the most influential parameters within the system. Specifically, $\beta _i$ contributes 81.9% to the variance of $I_{i}$, while $\beta _c$ contributes 83.6% to the variance of $I_{c}$. When considering the combined effect of $I_{i}$ and $I_{c}$, $\beta _i$ and $\beta _c$ contribute 31.8% and 49.1%, respectively. Furthermore, marginal influences are observed, such as the effect of $\beta _c$ on the variance of $I_{i}$ contributing 1.2% and the effect of $\beta _i$ on the variance of $I_{c}$ contributing 0.5%, attributed to cross-immunity. Depending on the output variable of interest, $c$, $\Omega _c$, $\Omega _i$, $\alpha _{c}$, $\alpha _{i}$, $\rho _c$, $\rho _i$, $\gamma _{c}$, $\gamma _{i}$, $\sigma _c$, $\sigma _i$, $\phi _{c}$, $\phi _{i}$, $\tau _{c}$, $\tau _{i}$, $\eta ^i_{c}$ and $\eta ^c_{i}$ have minimal or no direct influence on either $I_i$ or $I_c$, but their interactions with other parameters can significantly amplify their impact. In contrast, $\Lambda$, $\mu$, $\delta _{c}$, and $\delta _{i}$ show no direct influence on $I_i$, $I_c$, or $I_i + I_c$, and lack significant interactions with other parameters. Thus, they can be identified as common noninfluential factors within the model.

**Table 4:** Sensitivity indices for two output variables, $I_i$ and $I_c$, both individually and collectively, with respect to various input factors. The first-order Sobol index ${\textbf {SI}}_{st}^{P_{i}}$ reflects the direct contribution of $P_i$ to the variance of the output, while the total-order Sobol index ${\textbf {SI}}_{tot}^{P_{i}}$ measures the overall impact of $P_i$, including its interactions with other factors. ${\textbf {OVI}}$ represents the output variable of interest, and ${\textbf {I}}^{P_{i}} = {\textbf {SI}}_{tot}^{P_{i}} - {\textbf {SI}}_{st}^{P_{i}}$

Input factors	OVI= $I_{i}$			OVI = $I_{c}$			OVI = $(I_{i}+I_{c})$		
	${SI}_{st}^{P_{i}}$	${SI}_{tot}^{P_{i}}$	${I}^{P_{i}}$	${SI}_{st}^{P_{i}}$	${SI}_{tot}^{P_{i}}$	${I}^{P_{i}}$	${SI}_{st}^{P_{i}}$	${SI}_{tot}^{P_{i}}$	${I}^{P_{i}}$
$P_{1}= c$	0.004	0.044	0.040	0.002	0.034	0.032	0.004	0.046	0.041
$P_{2}= \Lambda$	0.000	0.000	0.000	0.000	0.000	0.000	0.000	0.000	0.000
$\vdots = \mu$	0.000	0.000	0.000	0.000	0.000	0.000	0.000	0.000	0.000
$\Omega _c$	0.000	0.000	0.000	0.000	0.002	0.003	0.000	0.001	0.002
$\Omega _i$	0.001	0.008	0.007	0.000	0.000	0.000	0.000	0.003	0.003
$\beta _c$	0.012	0.086	0.074	0.836	0.936	0.101	0.491	0.600	0.108
$\beta _i$	0.819	0.932	0.113	0.005	0.046	0.041	0.318	0.408	0.090
$\alpha _{c}$	0.000	0.000	0.000	0.000	0.006	0.006	0.000	0.003	0.003
$\alpha _{i}$	0.000	0.007	0.007	0.000	0.000	0.000	0.000	0.002	0.002
$\rho _c$	0.002	0.015	0.013	0.023	0.062	0.039	0.016	0.047	0.031
$\rho _i$	0.013	0.042	0.029	0.000	0.020	0.020	0.008	0.036	0.028
$\gamma _{c}$	0.000	0.001	0.001	0.005	0.014	0.009	0.003	0.008	0.005
$\gamma _{i}$	0.002	0.006	0.004	0.000	0.001	0.001	0.001	0.003	0.002
$\sigma _{c}$	0.001	0.001	0.000	0.005	0.013	0.008	0.004	0.008	0.004
$\sigma _{i}$	0.000	0.001	0.001	0.000	0.000	0.000	0.001	0.001	0.000
$\delta _{c}$	0.000	0.000	0.000	0.000	0.000	0.000	0.000	0.000	0.000
$\delta _{i}$	0.000	0.000	0.000	0.000	0.000	0.000	0.000	0.000	0.000
$\phi _{c}$	0.000	0.001	0.001	0.002	0.009	0.007	0.001	0.005	0.004
$\phi _{i}$	0.003	0.011	0.008	0.000	0.001	0.001	0.001	0.005	0.004
$\tau _{c}$	0.000	0.005	0.005	0.000	0.000	0.000	0.000	0.002	0.002
$\tau _{i}$	0.000	0.000	0.000	0.000	0.010	0.010	0.000	0.005	0.005
$\vdots = \eta ^i_{c}$	0.005	0.038	0.033	0.000	0.001	0.001	0.002	0.015	0.013
$P_{23}= \eta ^c_{i}$	0.000	0.002	0.002	0.001	0.025	0.024	0.001	0.015	0.014
Sum	0.861	1.198	0.337	0.877	1.180	0.303	0.849	1.211	0.362

**Fig. 9:**
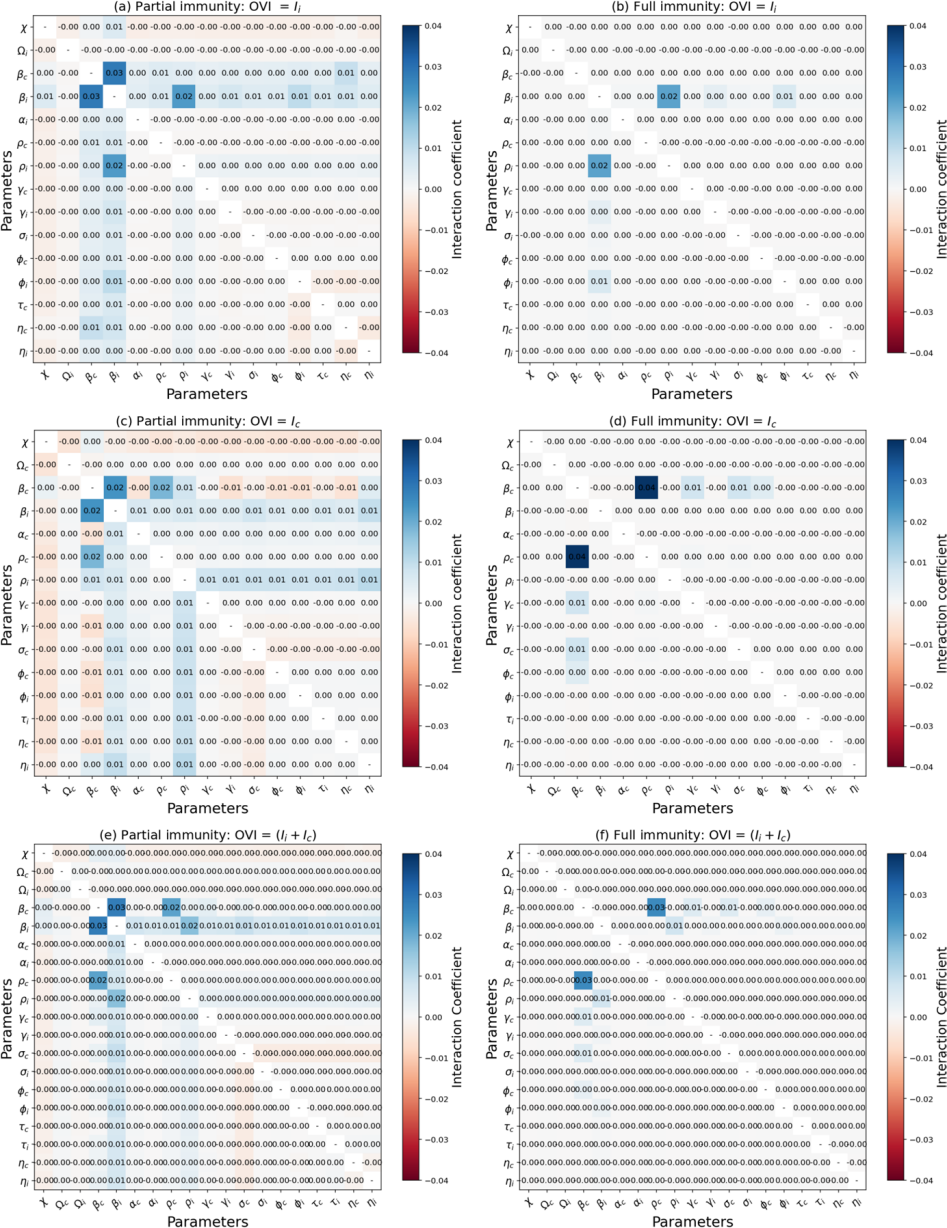
Visualization of parameter interactions in infection output variance using a symmetric matrix. Parameters with no interaction, as indicated in Table 4, have been omitted. ‘-’ indicates no self-interaction, while positive (negative) coefficients denote positive (inverse) interactions between parameters and output variance. OVI refers to the output variable of interest

In Fig. 9, we show the interaction between two input factors that influence the outputs $I_i$, $I_c$, and $I_i + I_c$. In particular, the interaction between $\beta _i$ and $\beta _c$ remains significant, with values of 0.03 and 0.02, particularly in the case of partial cross-immunity, as shown in subplots (a), (c) and (e). Positive values indicate that simultaneous increases in $\beta _i$ and $\beta _c$ result in higher levels of $I_i$, $I_c$, and $I_i + I_c$. However, in subplots (b), (d), and (f), which assume full cross-immunity, no interaction is observed between $\beta _i$ and $\beta _c$, suggesting that the two factors no longer influence each other under these conditions. Furthermore, interactions such as $(\beta _i, \rho _i)$ and $(\beta _c, \rho _c)$ have a comparable significance to the first-order effects of $\beta _i$ and $\beta _c$, showing a substantial impact on the variance of the output. These interactions remain significant even under full cross-immunity conditions.

## Discussion

This study investigates the dynamics of coexistence of influenza and SARS-CoV-2 using a mathematical model incorporating age-specific parameters. By performing sensitivity analyzes and fitting the model to real-world data using sampled parameter sets, the study explores transmission patterns and susceptibility variations between different age groups and pandemic waves. The results highlight the importance of age-specific factors in understanding disease dynamics and provide insights into the impact of interactions between key parameters on disease transmission.

Sensitivity analysis emphasizes the critical influence of $\beta _i$ and $\beta _c$ on the dynamics of influenza ($I_i$) and COVID-19 ($I_c$) infections, respectively, highlighting their substantial role in explaining variations in infection levels and underscoring their importance in understanding disease spread. Furthermore, the complexity of the model is increased by the effects of other parameters and their interactions, with some factors having minimal direct impact but potentially magnifying their influence through interactions with key parameters. In particular, the interaction between influenza ($\beta _i$) and SARS-CoV-2 ($\beta _c$) transmission rates remains a key determinant, especially in scenarios involving partial cross-immunity. This aligns with findings from Nuño et al. [17], who used a *SIRD* model to investigate the effects of partial cross-immunity on the spread of two cocirculating influenza pathogens. Their results suggested that partial cross-immunity could result in complex epidemic dynamics, including oscillations in the prevalence of each pathogen and potential co-existence. Fome et al. [37] found that robust cross-immunity, where exposure to one pathogen provides strong protection against the other, reduces susceptibility to the second pathogen, leading to a dominant peak reflecting the primary outbreak. However, weak cross-immunity increases the likelihood of co-existence of multiple pathogens, potentially resulting in overlapping or merged peaks. These findings suggest that when there is some degree of immune overlap between diseases, the interaction between their transmission rates becomes a crucial factor in determining the overall dynamics of infection.

Our model fitting to real data revealed that the age-structured model effectively captured the dynamics of both influenza and COVID-19, surpassing the combined model of all age groups (see Fig. 3). This underscores the importance of integrating age-related factors into epidemic modeling, given their significant influence on disease transmission dynamics, severity patterns, and long-term trends. When comparing the age-structured single and multi-pathogen models (Table 3), notable differences emerged in the estimated probability of infection ($\beta _i$ and $\beta _c$). For example, in the age group 0-4, the median value of $\beta _i$ was lower in the multi-pathogen model ($6.2 \times 10^{-12}$) compared to the mean value in the single pathogen model ($1.5 \times 10^{-4}$), while for $\beta _c$, the median in the multi-pathogen model ($0.0009$) significantly exceeded the mean in the single pathogen model ($0.00028$). These disparities between age groups indicate that the incorporation of multiple pathogens leads to notable differences in estimated transmission rates, likely due to complex interactions between pathogens and their impacts on transmission dynamics.

With regard to age-group transmission dynamics, both pathogens demonstrate varying transmission rates between different age groups. In the case of influenza, the age group 05-14 consistently emerges with the highest transmission potential, followed by the age groups 35-59 and 60-79 (see Figs. 5 and 6). This implies a significant role for younger people in influenza transmission, aligning with prior research such as [2, 66, 67]. These studies suggest that children and adolescents, particularly those in educational or childcare settings, serve as potential vectors for influenza transmission due to heightened social interactions. In addition, older adults, especially those aged 60 and older, face elevated risks of serious influenza complications [66].

In contrast, the transmission dynamics of COVID-19 shows higher reproduction numbers across all age groups compared to influenza, regardless of the time periods without (before December 27, 2020) and with (from December 27, 2020) COVID-19 vaccination and only periods with influenza vaccination. The 35-59 age group consistently shows the highest transmission potential, followed by the 15-34 and 60-79 age groups, as shown in Fig. 5. This underscores the critical role of middle-aged adults in the spread of COVID-19, corroborating findings from studies such as [27, 68], which highlight the substantial contribution of contacts to transmission, primarily within young adult and adult demographics.

## Conclusion

By integrating experimental data on SARS-CoV-2 and influenza, our study has been able to shed light on the potential for coexistence with competing viruses. It explained how the spread of COVID-19 and the flu varies between age groups. These changes in transmission rates between age groups indicate potential changes in susceptibility or behavior to these viruses. By dividing the population into different age groups, the accuracy and robustness of our model has been greatly improved. This distinction allowed for a more in-depth examination of the relationships between demographics and disease outcomes, providing valuable information on the mechanisms at work. We have elucidated the relationship between transmission dynamics, especially in populations with two circulating pathogens, through sensitivity analysis. The interaction between $\beta _c$ and $\beta _i$ shows the need for integrated approaches to manage and control infectious diseases and emphasizes the importance of assessing the combined effects of interventions that target one pathogen based on the transmission dynamics of another pathogen.

Although the single-strain model may show a better statistical fit in certain cases, the multi-pathogen model remains the preferred approach as it better reflects the real-world complexity of co-circulating pathogens. It incorporates crucial dynamics such as strain interaction, competition, and co-existence, factors essential for comprehensive epidemic forecasting and informed public health decision making. Unlike the single-strain framework, the multi-pathogen model enables a more nuanced evaluation of intervention and vaccination strategies by accounting for inter-strain effects. Even if this model occasionally yields a lower fit quality, it offers a more realistic and actionable depiction of disease behavior, particularly in scenarios involving simultaneous pathogen circulation.

Nevertheless, our models still face limitations in fully capturing the intricacies of co-infection and cross-immunity, underlining the need for further research. Although co-infections with SARS-CoV-2 and other respiratory viruses are reported to be uncommon [38], rare cases, such as the documented co-infection of SARS-CoV-2 and influenza A in a 4-month-old infant in Germany [39] demonstrate the importance of accurate diagnostics. Enhancing data collection, particularly in settings with higher co-infection rates, along with improving diagnostic techniques, can help uncover hidden patterns. These advancements will be critical for better understanding the epidemiological impact of co-infections and guiding the development of more effective, targeted public health interventions.

Cross-immunity has been among the areas of our focus in this study, yet more research is needed, particularly regarding age-specific partial cross-immunity. For example, [69] found that adults generally exhibit a stronger antibody response to SARS-CoV-2 compared to children, who show reduced protective responses. This variation implies that adults may possess stronger cross-immunity than children, making the latter more susceptible to co-circulating pathogens. Future models should incorporate age-specific cross-immunity parameters; in our model, where 0 signifies full protection while 1 means no protection, children could be assigned higher cross-immunity values, while adults could be assigned lower values reflecting their stronger immunity. Investigating the strength and duration of immunity across different age groups will enhance the refinement of vaccination and intervention strategies.

Although our model assumes that disease-induced deaths are rare, reflecting effective interventions that reduce mortality, we recognize that this simplification may not adequately account for the increased risks faced by specific age groups. Previous research indicates that people 65 years and older, as well as children under 5, are at increased risk of serious complications and death from influenza [2, 34]. Similarly, studies during the COVID-19 pandemic have shown that older adults and young children are significantly more vulnerable to severe outcomes, with higher rates of hospitalization and mortality reported among the elderly [33]. These limitations highlight critical areas for future research and emphasize the need for caution when interpreting our results in the context of these constraints.

## Supplementary Information

Supplementary Material 1.

## Data Availability

Data supporting the findings of this study are available from the authors upon reasonable request.
