# Supplementary material for: The interplay of influenza and COVID-19 in Germany, January 2020 - December 2022: a study of competitive disease dynamics with quarantine measures and partial cross-immunity
Source: BMC Public Health. 2025 Sep 9;25:3044. doi: 10.1186/s12889-025-24362-z (PMC12418641; doi:10.1186/s12889-025-24362-z)
Supplement: Supplementary file 1 — Supplementary Material 1. [file 12889_2025_24362_MOESM1_ESM.pdf]

The Interplay of Influenza and COVID-19 in Germany, January 2020 - December 2022: A Study of Competitive Disease Dynamics with Quarantine Measures and Partial Cross-Immunity

Anna Daniel Fome[1,2], Isti Rodiah[3], Wolfgang Bock[4*], Berit Lange[3,5], Axel Klar[1]

[1]Department of Mathematics, Rheinland-Pfälzische Technische Universität Kaiserslautern-Landau, Gottlieb-Daimler-Str.48, Kaiserslautern, 67663, Germany.
[2]Department of Economics, Mathematics and Statistics, Jordan University College, P.O.Box 1878, Morogoro, Tanzania.
[3]Department of Epidemiology, Helmholtz Centre for Infection Research (HZI), Inhoffenstr. 7, Braunschweig, 38124, Germany.
[4*]Department of Mathematics, Linnaeus University, Universitetsplatsen 1, Växjö, 35252, Sweden.
[5]German Centre for Infection Research (DZIF), Inhoffenstr. 7, Braunschweig, 38124, Germany.

*Corresponding author(s). E-mail(s): wolfgang.bock@lnu.se;
Contributing authors: fome@mathematik.uni-kl.de;
Isti.Rodiah@helmholtz-hzi.de; Berit.Lange@helmholtz-hzi.de;
klar@mathematik.uni-kl.de;

Keywords: Influenza, COVID-19, Age-stratified model, Multi-pathogen, Competition, Co-existence, Cross-immunity

Supplementary Information

Appendix A Model Diagram

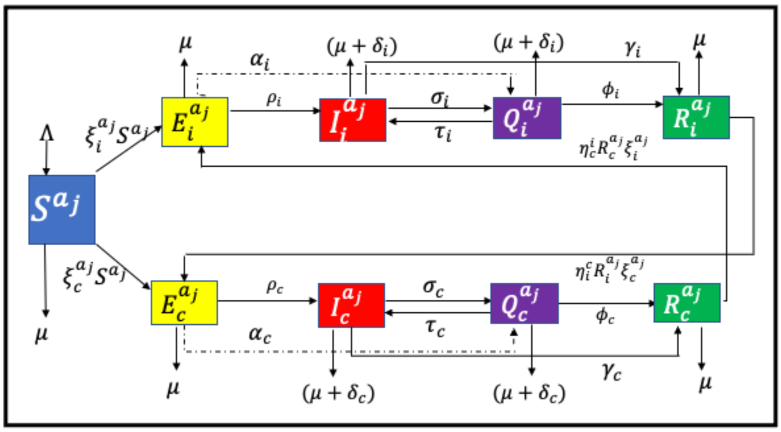

Fig. A1 Flowchart depicting the dynamics of a two-strain system for age group 1 ($a_{j=1}$). Similar structures are employed for $j = 2, 3, \cdots, 6$, with the exception that there is no inflow rate into the susceptible group.

Figure A1 presents an age-structured compartmental model that describes the cocirculation of two respiratory pathogens, pathogen-i (influenza) and pathogen-c (SARS-CoV-2), within an age group a_j. More details are available in Section 2.2 of the main manuscript.

Appendix B Social Contact Matrix and Mixing Coefficients

The matrix $M_{a_{jl}}$ quantifies the average daily contacts between individuals in the age group a_j (contacting group) and a_l (contacted group), based on the POLYMOD study for Germany [1]. This matrix has been adapted to match the six age classes used in the model.

The normalized mixing coefficients used in Equation (3) in the main manuscript are given by:

$$\chi_k^{a_{jl}} = \frac{M_{a_{jl}}}{N^{a_j}}, \quad \text{where } k \in \{i, c\}, \tag{B1}$$

where N^{a_j} is the total population in age group a_j. The full matrix is as follows:

$$M_{a_{jl}} = \begin{bmatrix} & \textbf{0–4} & \textbf{5–14} & \textbf{15–34} & \textbf{35–59} & \textbf{60–79} & \textbf{80+} \\ \textbf{0–4} & 1.90 & 0.45 & 0.85 & 0.75 & 0.13 & 0.07 \\ \textbf{5–14} & 1.15 & 4.19 & 1.11 & 1.87 & 0.29 & 0.61 \\ \textbf{15–34} & 2.80 & 1.77 & 5.75 & 2.82 & 0.93 & 1.41 \\ \textbf{35–59} & 2.40 & 2.58 & 3.50 & 4.61 & 2.44 & 2.08 \\ \textbf{60–79} & 0.46 & 0.41 & 1.14 & 1.92 & 1.71 & 0.87 \\ \textbf{80+} & 0.38 & 0.87 & 0.82 & 1.50 & 1.67 & 0.76 \end{bmatrix} \tag{B2}$$

Appendix C Derivation of Age-specific Control Reproduction Numbers

To compute the control reproduction number $\tilde{\mathcal{R}}^q$ for model (2) in the main manuscript, we apply the next generation matrix method as described in [2]. The infected compartments considered are $E_i^{a_j}, E_c^{a_j}, I_i^{a_j}, I_c^{a_j}, Q_i^{a_j}, Q_c^{a_j}$ for each age group $j = 1, 2, \cdots, 6$. We express the model in the form $X' = \mathcal{F} - \mathcal{V}$, where $\mathcal{F}$ contains new infection terms, and $\mathcal{V}$ represents transitions between compartments. The Jacobians F and V of these terms are evaluated at disease-free equilibrium (DFE), and the next-generation matrix is constructed as $\mathbf{K} = FV^{-1}$. The spectral radius of $\mathbf{K}$ gives the basic reproduction number. For each age group a_j, the age-specific quarantine reproduction numbers for COVID-19 and influenza are given by:

$$\begin{aligned} \tilde{\mathcal{R}}_c^{a_j} &= \left(\frac{\beta_c^{a_j}(t)\chi^{a_{jl}}(t)(\alpha_c \mathbf{q}_c + \rho_c\sigma_c + \alpha_c\tau_c + \mathbf{r}_c\rho_c)}{\mathbf{p}_c((\gamma_c + \delta_c + \mu)\mathbf{r}_c + (\phi_c + \delta_c + \mu)\sigma_c)} \right) S^{a_j}(t), \\ \tilde{\mathcal{R}}_i^{a_j} &= \left(\frac{\beta_i^{a_j}(t)\chi^{a_{jl}}(t)(\alpha_i \mathbf{q}_i + \rho_i\sigma_i + \alpha_i\tau_i + \mathbf{r}_i\rho_i)}{\mathbf{p}_i((\gamma_i + \delta_i + \mu)\mathbf{r}_i + (\phi_i + \delta_i + \mu)\sigma_i)} \right) S^{a_j}(t) \end{aligned} \tag{C3}$$

where:

$$\begin{aligned} \mathbf{p}_i &= \alpha_i + \rho_i + \mu, & \mathbf{q}_i &= \gamma_i + \sigma_i + \delta_i + \mu, & \mathbf{r}_i &= \phi_i + \tau_i + \delta_i + \mu, \\ \mathbf{p}_c &= \alpha_c + \rho_c + \mu, & \mathbf{q}_c &= \gamma_c + \sigma_c + \delta_c + \mu, & \mathbf{r}_c &= \phi_c + \tau_c + \delta_c + \mu. \end{aligned}$$

These expressions allow us to estimate the impact of quarantine and isolation on transmission dynamics between different age groups.

Appendix D Breakdown of sensitivity evaluation methods

In practical applications, these analyses will be carried out using a computer program; thus, here we only describe the variance-based numerical procedure for computing indices.

Step 1. Define the output function and the targeted parameters:
Using the notation introduced in [3, 4], we denote the output function of interest as

$$Y = g(P_1, P_2, \cdots, P_k), \tag{D4}$$

over the domain

$$\Gamma = [0,1]^k = \{(P_1, P_2, \cdots, P_k) | 0 \leq P_i \leq 1 \text{ for } i = 1, 2, \cdots, k\},$$

which represents the k-dimensional unit hypercube. P_i where $i = 1, 2, \cdots, k = 23$ are input factors, each of which is uniformly distributed within the specified ranges, as indicated in Table 2 in the main manuscript. For each range of variation P_i, we want to determine what would happen to the uncertainty of Y if we could fix factors.

We define a function Y as an integral over time based on the model output of interest. Sobol's method expands g in terms of increasing dimensions:

$$g = g_0 + \sum_i g_i + \sum_i \sum_{j>i} g_{ij} + \cdots + g_{12\cdots k}.$$

Each term in the expansion is square-integrable in its domain and depends only on the factors specified by its index. For example,

$$g_i = g_i(P_i), \quad g_{ij} = g_{ij}(P_i, P_j),$$

and so on, resulting in a total of 2^k terms, including the baseline value g_0. Here, g_i represents the contribution of factor P_i, while g_{ij} captures the contribution of the interaction between P_i and P_j. Given $Y = g(P)$, the total variance $V(Y)$ can be decomposed as:

$$V(Y) = \sum_{i=1}^{l} V_i + \sum_{i<j} V_{ij} + \cdots + V_{12\cdots k}$$

where: V_i is the variance contribution of parameter P_i (first-order effect). V_{ij} is the variance contribution due to the interaction between the parameters P_i and P_j (second order effect). Higher-order terms represent interactions among more parameters.

Step 2. Sample generation:
We start by generating a sample matrix of random numbers with dimensions $(N, 2k)$, where k represents the number of input parameters. Next, we split this sample matrix into two main sample matrices, $\mathbf{A}$ and $\mathbf{B}$, each of size $N \times k$. These matrices contain half of the samples each. We then define a matrix $\mathbf{B}_{\mathbf{A}}^{(i)}$, where each column is taken from matrix $\mathbf{B}$ except for the i-th column, which is replaced with the corresponding column from matrix $\mathbf{A}$. This results in a set of matrices, each representing a specific interaction scenario between the parameters of matrices $\mathbf{A}$ and $\mathbf{B}$.

Step 3. Computation of the model output:

For each set of input values in the sample matrices $\mathbf{A}$, $\mathbf{B}$, and $\mathbf{B}_{\mathbf{A}}^{(i)}$, we compute the corresponding model outputs. This results in three vectors of model outputs, each of dimension $N \times 1$:

$$Y_{\mathbf{A}} = g(\mathbf{A}), \quad Y_{\mathbf{B}} = g(\mathbf{B}), \quad Y_{\mathbf{B}_{\mathbf{A}}^{(i)}} = g(\mathbf{B}_{\mathbf{A}}^{(i)})$$

These vectors represent the model outputs for the samples in matrices $\mathbf{A}$, $\mathbf{B}$, and the modified matrix $\mathbf{B}_{\mathbf{A}}^{(i)}$, respectively.

Step 4. Estimation of sensitivity indices:

First-order Sobol index $\mathbf{SI}_{st}^{P_i}$: Represents the contribution of P_i alone to the output variance. It is defined as:

$$\mathbf{SI}_{st}^{P_i} = \frac{V(E(Y|P_i))}{V(Y)} = \frac{\frac{1}{N}\sum_{i=1}^{N} Y_{\mathbf{A}}^{(i)} Y_{\mathbf{B}_{\mathbf{A}}^{(i)}}^{(i)} - g_0^2}{\frac{1}{N}\sum_{i=1}^{N} (Y_{\mathbf{A}}^{(i)})^2 - g_0^2},$$

where g_0 is calculated as: $g_0 = \frac{1}{N}\sum_{i=1}^{N} Y_{\mathbf{A}}^{(i)}$. Here, $V(E(Y|P_i))$ denotes the variance of the conditional expectation of Y given P_i, taking into account the variability in Y that is explained solely by P_i. Total order Sobol index $\mathbf{SI}_{tot}^{P_i}$: Quantifies the contribution of P_i, including all its interactions with other variables. It is expressed as:

$$\mathbf{SI}_{tot}^{P_i} = 1 - \frac{V(E(Y|P_{\sim i}))}{V(Y)} = \frac{\frac{1}{N}\sum_{i=1}^{N} Y_{\mathbf{B}}^{(i)} Y_{\mathbf{B}_{\mathbf{A}}^{(i)}}^{(i)} - g_0^2}{\frac{1}{N}\sum_{i=1}^{N} (Y_{\mathbf{A}}^{(i)})^2 - g_0^2}.$$

Here, $V(E(Y|P_{\sim i}))$ represents the variance in Y that is not explained by P_i, accounting for the exclusion of its direct and interaction effects.

Step 5. Simulating the model:

At this stage, we calculate sensitivity indices to assess how individual parameters, their collective impact, and their interactions contribute to the variability observed in the model's results. To generate combinations of parameters, we used Saltelli sampling, leveraging the *saltelli.sample* function provided by the SALib library [5, 6]. Model simulations were conducted using the Python programming language (version 3.10.11) using the *sobol.analyze* function. The sample size, denoted as N, was set to 2^{15}. Each simulation run took approximately 0.001 seconds to complete. The total computational cost of executing the model for $k = 23$ factors amounted to $(2k + 2)N$. A summary of the results obtained is presented in Table 4 (main manuscript), and visual representations are provided in Figure 9 (main manuscript).

References

[1] Mossong, J., Hens, N., Jit, M., Beutels, P., Auranen, K., Mikolajczyk, R., Massari, M., Salmaso, S., Tomba, G.S., Wallinga, J., *et al.*: Social contacts and mixing

patterns relevant to the spread of infectious diseases. PLoS medicine **5**(3), 74 (2008)

[2] Martcheva, M.: An Introduction to Mathematical Epidemiology vol. 61. Springer, Heidelberg (2015). https://link.springer.com/book/10.1007/978-1-4899-7612-3

[3] Saltelli, A., Annoni, P., Azzini, I., Campolongo, F., Ratto, M., Tarantola, S.: Variance based sensitivity analysis of model output. design and estimator for the total sensitivity index. Computer physics communications **181**(2), 259–270 (2010)

[4] Andrea, S., Marco, R., Terry, A., Francesca, C., Jessica, C., Debora, G., Michaela, S., Stefano, T.: Global sensitivity analysis: the primer. Ist ed., John Wiley & Sons, The Atrium, Southern Gate, Chichester, England (2008)

[5] Herman, J., Usher, W.: Salib: An open-source python library for sensitivity analysis. Journal of Open Source Software **2**(9), 97 (2017)

[6] Oliphant, T.E.: Python for scientific computing. Computing in science & engineering **9**(3), 10–20 (2007) https://doi.org/10.1109/MCSE.2007.58
